# Exhibition of antifungal resistance by sterol-auxotrophic strains of *Candida glabrata* with intact virulence

**DOI:** 10.1093/jacamr/dlac018

**Published:** 2022-03-07

**Authors:** Minoru Nagi, Koichi Tanabe, Kazuko Tanaka, Keigo Ueno, Hironobu Nakayama, Jun Ishikawa, Masahiro Abe, Satoshi Yamagoe, Takashi Umeyama, Shigeki Nakamura, Motoyuki Sugai, Kevin C. Hazen, Yoshitsugu Miyazaki

**Affiliations:** 1 Department of Fungal Infection, National Institute of Infectious Diseases, 1-23-1 Toyama, Shinjuku-ku, Tokyo 162-8640, Japan; 2 Antimicrobial Resistance Research Center, National Institute of Infectious Diseases, 1-23-1 Toyama, Shinjuku-ku, Tokyo 162-8640, Japan; 3 Department of Food Science and Human Nutrition, Faculty of Agriculture, Ryukoku University, 1-5 Yokotani, Seta Oe-cho, Otsu, Shiga 520-2194, Japan; 4 Research Center for Fermentation and Brewing, Ryukoku University, 1-5 Yokotani, Seta Oe-cho, Otsu, Shiga 520-2194, Japan; 5 Faculty of Pharmaceutical Sciences, Suzuka University of Medical Science, 3500-3 Minami-Tamagaki, Suzuka, Mie 513-8670, Japan; 6 Department of Microbiology, Tokyo Medical University, 6-1-1 Shinjuku, Shinjuku-ku, Tokyo 160-8402, Japan; 7 Department of Pathology, Duke University Health System, 2902 DUMC, 116 CARL Building, Durham, NC 27710, USA

## Abstract

**Background:**

*Candida glabrata* is an emerging fungal pathogen in immune-compromised hosts. Previously undetected *C. glabrata* isolates were successfully recovered from clinical specimens by adding sterols to the growth medium. The clinical isolates are unable to synthesize ergosterol but can take up exogenous sterols under aerobic conditions.

**Objectives:**

This study characterizes the sterol-auxotrophic *C. glabrata* strains, examines the mutation(s) in sterol synthesis genes, characterizes the drug susceptibility and evaluates the virulence in a mouse infection model.

**Methods:**

Drug susceptibility of the *C. glabrata* strains was evaluated in a sterol-supplemented medium. The coding sequences of the sterol synthesis genes were analysed in six sterol-auxotrophic strains of *C. glabrata*. The fungal burden of mice infected with *C. glabrata* strain was determined.

**Results:**

The sterol-auxotrophic strains showed high-level resistance to both azoles and amphotericin B when sterols were supplied in the test medium. Additionally, the strains harbour missense mutations in either *ERG1* or *ERG7*. Significant differences in fungal burden were not observed between the sterol-auxotrophic strain and the sterol-competent strain with the mice infection models.

**Conclusions:**

The sterol-auxotrophic *C. glabrata* strain investigated in this study seemed to maintain intact virulence, probably due to the supply of exogenous sterols from host organ(s). This suggests that exogenous sterol uptake develops antifungal resistance during infection.

## Introduction

The most common species that causes candidiasis has been *Candida albicans*. An emerging trend of a shift toward infections with non-albicans *Candida* species has been observed in candidiasis.^1^ Consequently, *Candida glabrata* has increased as an agent of bloodstream infection, especially within ICUs of some hospitals. The presence of *C. glabrata* as an aetiological agent presents with some challenges in patient management because of its potentially reduced susceptibility to azole antifungals. In a global surveillance study, approximately 18.3% of all *C. glabrata* clinical isolates were found to be fluconazole resistant (MIC of >64 mg/L).^2,3^

*C. glabrata* strains that are unable to synthesize ergosterol have been isolated from the urinary tract,^4,5^ indicating that endogenous ergosterol synthesis of *C. glabrata* could be omitted inside the host tissues. The most probable hypothesis is that exogenous sterol uptake can replace endogenous ergosterol synthesis for the growth of *C. glabrata* during an infection.

*C. glabrata* closely resembles *Saccharomyces cerevisiae* compared with other *Candida* spp. in several of its features. *S. cerevisiae* can accumulate exogenous sterol under anaerobic or aerobic conditions when harbouring specific mutations. Anaerobic sterol uptake has also been observed in *C. glabrata*,^6^ and it has been estimated that similar sterol uptake mechanisms are conserved between these genetically close relatives. Our past studies have demonstrated that a sterol transporter (Aus1),^6,7^ two transcription factors (Upc2A and Upc2B)^8^ and a putative mannoprotein (Tir3)^9^ are responsible for sterol uptake in yeast. The mutant *C. glabrata*, which lacks *AUS1*, exhibited reduced virulence in mouse infection experiments.^6^ We also showed that the serum, as an exogenous sterol source, promoted both exogenous sterol uptake and the growth of *C. glabrata* cells, wherein endogenous sterol synthesis was inhibited by azole treatment.^6,10,11,12^ These observations strongly suggest that sterol uptake plays a significant role in the infection and azole resistance of *C. glabrata*.

Herein, we characterized the antifungal susceptibility, sterol uptake activity and virulence of sterol-auxotrophic clinical isolates of *C. glabrata*. In addition, the expression levels of the genes responsible for sterol uptake and the sequences of ergosterol synthesis genes were also determined with these strains. These results altogether indicate that endogenous sterol synthesis in *C. glabrata* cells is unnecessary during an infection. The sterol uptake activity of *C. glabrata* induces tolerance to azoles during antifungal chemotherapy.

## Materials and methods

### Ethics

All animal experiment protocols complied with the guidelines and policies of the Principles of Morality for Animal Experiments of the National Institute of Infectious Disease, Japan (approval number 111073).

### Strains and media

The *C. glabrata* strains used in this study are listed in Table 1. Yeasts were grown in yeast extract–peptone–dextrose (YPD) medium [1% Bacto yeast extract (Difco), 2% Bacto-peptone (Difco) and 2% glucose] containing 0.5% Tween 80-ethanol [1:1 (vol/vol)], and 20 mg/L sterols were supplied if indicated. Solid media were supplemented with 2% agar (Nacalai Tesque, Kyoto, Japan). The following compounds, ergosterol, ergosterol acetate and ergosterol propionate, were purchased from FUJIFILM Wako Pure Chemical Corporation. Ltd, Osaka, Japan. Lathosterol and β-sitosterol were obtained from Nagara Science Co. Ltd, Gifu, Japan and TAMA Biochemical Co. Ltd, Tokyo, Japan, respectively.

**Table 1.** Strains used in this study

Strain	Description	References
CBS138	Reference strain	CBS
L999^a^	Bile-independent clinical isolate	Hazen *et al.* (2005)^5^
73246	Bile-dependent clinical isolate	Hazen *et al.* (2005)^5^
S53452	Bile-dependent clinical isolate	Hazen *et al.* (2005)^5^
H32441	Bile-dependent clinical isolate	Hazen *et al.* (2005)^5^
M34736	Bile-dependent clinical isolate	Hazen *et al.* (2005)^5^
W16119	Bile-dependent clinical isolate	Hazen *et al.* (2005)^5^

a Putative parental strain of 73246.

The rest used for growth supplementation were obtained from Sigma–Aldrich. The growth rescue of sterol-auxotrophic isolates was evaluated by the simple streak assay. Each strain was streaked on the YPD agar medium containing 20 mg/L sterols and incubated at 37°C for 24 h. In this study, 20 mg/L of any sterol was enough to support the growth of the sterol-auxotrophic isolates either on agar medium or in liquid medium.

### Quantification of sterols by HPLC

Ergosterol, cholesterol or lanosterol uptake was detected by reverse-phase HPLC as described before.^6^ The mean values and standard deviation of triplicate measurements from a representative experiment are shown.

### Antifungal susceptibility

Approximately 2* *×* *10^3^ cells were used to inoculate YPD medium (200 μL) and were then cultured with serially diluted fluconazole (from 0 to 1024 mg/L; Pfizer), or amphotericin B (from 0 to 64 mg/L; Sigma–Aldrich Chemical Co., St Louis, MO, USA). After incubation at 37°C for 24 h, the OD at 595 nm of the culture was measured to determine antifungal susceptibility using a Beckman Coulter DTX880 Multimode Detector (Fullerton, CA, USA). No significant growth of *C. glabrata* cells was observed in RPMI1640.

### Semi-quantitative RT-PCR

Cells grown in YPD medium containing 20 mg/L cholesterol and 0.5% Tween 80-ethanol at 37°C overnight were inoculated at approximately 1* *×* *10^6^ cells/mL and cultured for 4 h in YPD medium in the presence of 20 mg/L cholesterol and 0.5% Tween 80-ethanol. Total RNA was prepared from the cells and semi-quantitative RT-PCR was performed as described elsewhere^6^ except for the following: SuperScript^™^ VILO^™^ cDNA Synthesis Kit (Invitrogen) and a Mx3000P qPCR System (Agilent Technologies, Santa Clara, CA, USA). All primers used for qRT-PCR are listed in Table S1 (available as Supplementary data at *JAC-AMR* Online). All experiments were repeated with three independent preparations of RNA, and a representative result was exhibited. The mean fold changes compared with the value of CBS138 and standard deviation of triplicate measurements from a representative experiment are shown.

### Sequencing of ergosterol biosynthesis genes

The ergosterol biosynthesis genes of the sterol-auxotrophic *C. glabrata* isolates other than 73246 were determined by Sanger sequencing. The cells were grown in a 1.5 mL YPD medium containing 20 mg/L cholesterol and 0.5% Tween 80-ethanol at 30°C overnight. Genomic DNA was isolated from an overnight culture using the Dr. GenTLE (from yeast) High Recovery kit (Takara-Bio, Otsu, Japan). The high-fidelity Phusion DNA polymerase (New England Biolabs Japan) was used for amplification. The PCR conditions involved 35 cycles of denaturation at 95°C for 30 s, annealing at 55°C for 10 s and extension at 72°C for 120 s. Purification of PCR fragments was performed by ExoSAP-IT^™^ PCR Product Cleanup (Life Technologies Corporation, Carlsbad, CA, USA) following the manufacturer’s instructions. The sequences of all amplified DNA fragments were determined using the BigDye Terminator v.3.1 Cycle Sequencing Kit (Life Technologies, Tokyo, Japan), and analysed using the 3730xl DNA Analyzer (Life Technologies) by a customized service (Premix sequence, Takara-bio). The primers for amplification and sequencing are listed in Table S1.

As to the 73246 alone, next-generation sequencing was performed using the Illumina paired-end technology. The trimmed reads were assembled with the Velvet 1.2.10.^13^ Mapping of the reads to a reference sequence was performed with BWA 0.7.17,^14^ and variant calling was performed with Platypus 0.8.1.1.^15^

### Animal studies

The fungal tissue burden and expression analysis were performed as described previously.^16^ Five male BALB/c mice aged 7 weeks (Japan SLC Inc.) were used for a group. To establish *C. glabrata* infection, mice were injected into their tail vein with saline suspensions of 1 × 10^7^ viable cells (in a volume of 200 μL). After 7 days, mice were sacrificed by CO_2_ inhalation, and target organs (kidney and spleen) were excised aseptically, individually weighed and homogenized in sterile saline using a 70 μm Cell strainer (BD Falcon, Franklin Lakes, NJ, USA). Organ homogenates were diluted and plated onto YPD containing streptomycin sulphate salt (Sigma–Aldrich), penicillin G sodium salt (Sigma–Aldrich), 20 mg/L cholesterol and 0.5% Tween 80-ethanol. Colonies were counted after 1 day of incubation at 37°C and the numbers of cfu/g of the organ were calculated. Statistical analyses were conducted using GraphPad Prism6^™^ via an unpaired *t*-test with a significance level of *P < *0.05.

## Results

### Exogenously supplied sterols were aerobically taken up in the sterol-auxotrophic strains

**Figure 1.**
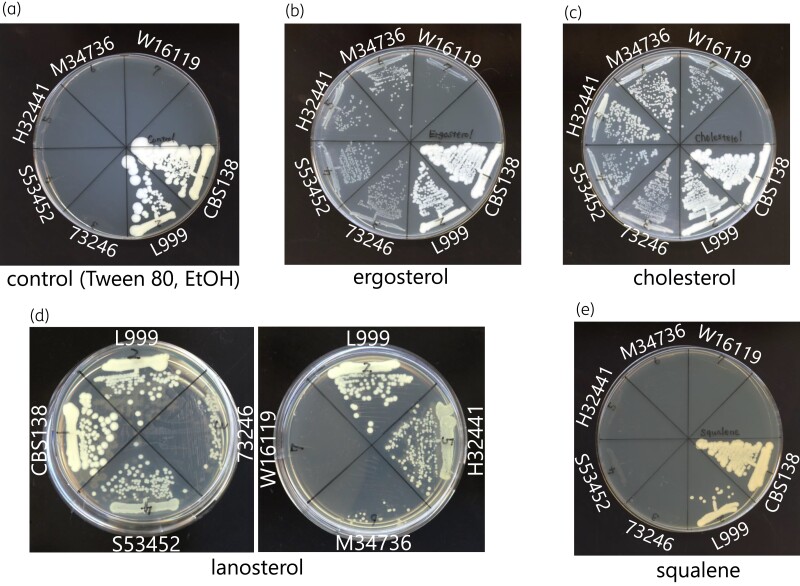
Growth restoration of the sterol-auxotrophic *C. glabrata* strains with authentic sterols. Each strain was streaked on YPD agar medium in the presence of 20 mg/L of ergosterol (b), cholesterol (c), lanosterol (d) or squalene (e) (a; vehicles). The plates were incubated at 37°C for 24 h. EtOH, ethanol.

**Figure 2.**
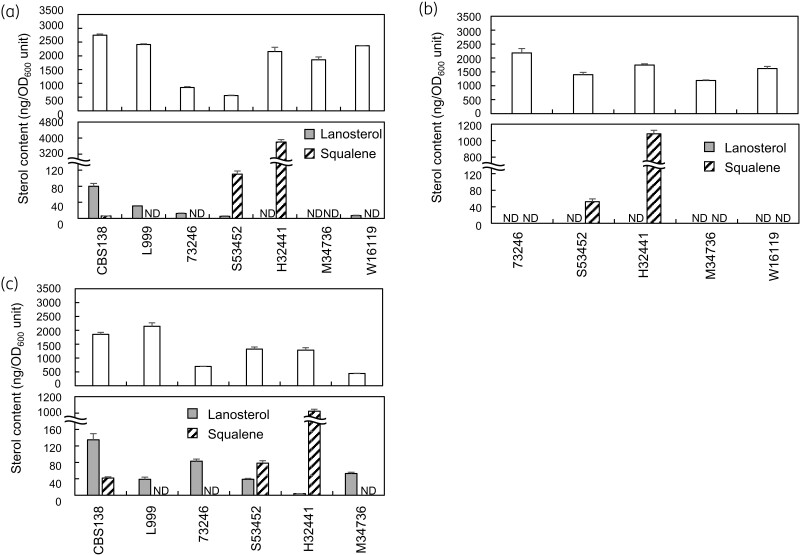
Sterol analysis of the sterol-auxotrophic *C. glabrata* strains. The sterol compositions of the *C. glabrata* cells were quantitatively determined. Ergosterol (a), cholesterol (b) or lanosterol (c) (final concentration of 20 mg/L for each) was exogenously supplied for growth. The upper panels show the amount of ergosterol (a and c) and cholesterol (b). The bottom panels are for lanosterol and squalene. The mean values and standard deviation of triplicate measurements from a representative experiment are shown.

The growth of these strains on YPD agar plates in various sterols and squalene, an intermediate compound in the sterol synthesis pathway, were examined (Figure 1). Cholesterol or ergosterol supported the growth of sterol-auxotrophic strains under aerobic conditions, as previously reported (Figure 1b and c). The taken-up sterols were detected in the strains, and the amounts of those in the cells except 73246 and S53452 were roughly comparable to ergosterol (∼2500 ng/OD_600_ unit) in the sterol-competent strains, CBS138 or L999 (Figure 2, Table S2). The amount of taken-up ergosterol in 73246 and S53452 was 41.3% (1025* *±* *43 ng/OD_600_ unit) and 23.4% (582* *±* *14 ng/OD_600_ unit) of that in the sterol-competent L999. The amount of taken-up total cholesterol in the strains ranged from 1301 (M34736) to 2335 (73246) ng/OD_600_ unit. These suggest that ergosterol, the final product of yeast sterol synthesis or alternative cholesterol directly compensates for the ergosterol shortage in the clinical isolates of *C. glabrata* cells. Exogenously supplied sterols were reported to be esterified immediately after being incorporated in *S. cerevisiae*.^17^ The comparison between the total (sum of esterified- and free-sterols) and free revealed that both ergosterol and cholesterol were retained as free form (Table S2).

Lanosterol also supported the growth of the mutants except for W16119 (Figure 1d). Two of them (S53452 and H32441) showed complete growth restoration with lanosterol, whereas the other two (73246 and M34736) exhibited only partial growth restoration (Figure 1d). A significant amount of ergosterol was detected in the strains with growth restoration by lanosterol (Figure 2c, Table S2). These results indicate that taken-up lanosterol was converted into ergosterol in these strains.

Additionally, to major sterols, 17 commercially available sterols and sterol esters were evaluated for rescuing the growth of sterol-auxotrophic strains (Table 2). Ten compounds (group A) could support the growth of all tested *C. glabrata* strains. 25-Hydroxycholesterol was unable to support the growth of any strain. The results of six of the compounds (group B) partially supported the growth depending on the strain.

**Table 2.** The growth profile of the sterol-deficient clinical isolates of *C. glabrata* in the presence of various sterols or sterol-related compounds

Supplements (20 mg/L)	Bile-dependent clinical isolates				
	73246	S53452	H32441	M34736	W16119
Ergosterol	+	+	+	+	+
Lanosterol	+	+	+	+	−
Lathosterol	+	+	+	+	+
7-Dehydrocholesterol	+	+	+	+	+
Ergosterol acetate	+	+	+	+	+
Ergosterol propionate	+	+	+	−	+
25-Hydroxycholesterol	−	−	−	−	−
Cholesterol	+	+	+	+	+
Stigmasterol	−	+	+	−	−
β-Sitosterol	−	+	+	−	−
Campesterol	+	+	+	+	+
Desmosterol	+	+	+	+	+
Brassicasterol	+	+	+	+	+
Fucosterol	−	+	+	−	+
β-Cholestanol	+	+	+	+	+
Stigmastanol	+	+	+	+	+
Cholesteryl acetate	+	+	+	−	+

+, visible growth in streak test with YPD medium supplemented by indicated compound; −, no growth in streak test with YPD medium supplemented by indicated compound.

### Exogenously supplied sterols affect the drug susceptibilities of the sterol-auxotrophic strains

Exogenous sterol also affected the drug susceptibility of the sterol-auxotrophic cells (Table 3). The strains exhibited extremely high MIC (more than 1024 mg/L, the maximum concentration in this assay) of fluconazole in the presence of any sterols that were categorized group A (Table 3).

**Table 3.** The fluconazole susceptibility of the sterol-deficient clinical isolates of *C. glabrata* in the presence of various sterols or sterol-related compounds

Supplements (20 mg/L)	Strains						
	CBS138	L999	73246	S53452	H32441	M34736	W16119
Tween 80 and ethanol (vehicle)	128	16	ND	ND	ND	ND	ND
Ergosterol	128	32	>1024	>1024	>1024	>1024	>1024
Lanosterol	128	16	>1024	>1024	>1024	>1024	ND
Lathosterol	128	16	>1024	>1024	>1024	>1024	>1024
7-Dehydrocholesterol	128	32	>1024	>1024	>1024	>1024	>1024
Ergosterol acetate	128	16	>1024	>1024	>1024	>1024	>1024
Ergosterol propionate	128	16	>1024	>1024	ND	ND	>1024
25-Hydroxycholesterol	128	16	ND	ND	ND	ND	ND
Cholesterol	128	16	>1024	>1024	>1024	>1024	>1024
Stigmasterol	128	16	ND	>1024	>1024	ND	ND
β-Sitosterol	128	16	ND	>1024	>1024	>1024	ND
Campesterol	128	16	>1024	>1024	>1024	>1024	>1024
Desmosterol	128	16	>1024	>1024	>1024	>1024	>1024
Brassicasterol	128	16	>1024	>1024	>1024	>1024	>1024
Fucosterol	128	16	ND	>1024	ND	ND	>1024
β-Cholestanol	128	16	>1024	>1024	>1024	>1024	>1024
Stigmastanol	128	16	>1024	>1024	>1024	>1024	>1024
Cholesteryl acetate	128	16	>1024	>1024	ND	ND	>1024

ND, not determined due to poor or no growth in liquid medium.

The addition of cholesterol but not ergosterol increased the MIC of amphotericin B (Table 4). The sterol-mediated alteration of antifungal susceptibility was not observed in the sterol-competent strains. These results indicated that exogenous sterol was aerobically taken up, and it is sorted to the plasma membrane in the sterol-auxotrophic strains.

**Table 4.** The amphotericin B susceptibility of the sterol-deficient clinical isolates of *C. glabrata* in the presence of various sterols or sterol-related compounds

Supplements (20 mg/L)	Strains						
	CBS138	L999	73246	S53452	H32441	M34736	W16119
No reagent	0.5	0.25	ND	ND	ND	ND	ND
Tween 80 and ethanol (vehicle)	8	4	ND	ND	ND	ND	ND
Ergosterol	4	2	1	2	2	2	0.5
Lanosterol	16	8	8	8	8	8	ND
Lathosterol	8	4	4	8	8	8	4
7-Dehydrocholesterol	4	2	2	8	4	4	4
Ergosterol acetate	8	4	4	8	4	8	4
Ergosterol propionate	8	4	2	8	ND	ND	2
25-Hydroxycholesterol	4	2	ND	ND	ND	ND	ND
Cholesterol	8	4	16	>64	16	>64	16
Stigmasterol	4	2	ND	8	16	ND	ND
β-Sitosterol	8	4	ND	16	32	16	ND
Campesterol	8	4	16	32	16	>64	16
Desmosterol	8	8	16	16	16	16	16
Brassicasterol	4	2	4	16	8	16	16
Fucosterol	8	4	ND	16	ND	ND	16
β-Cholestanol	8	4	16	16	32	16	4
Stigmastanol	8	2	8	32	64	>64	8
Cholesteryl acetate	8	4	16	>64	ND	ND	64

ND, not determined due to poor or no growth in liquid medium.

### Aerobic upregulation of sterol uptake genes in the sterol-auxotrophic strains

**Figure 3.**
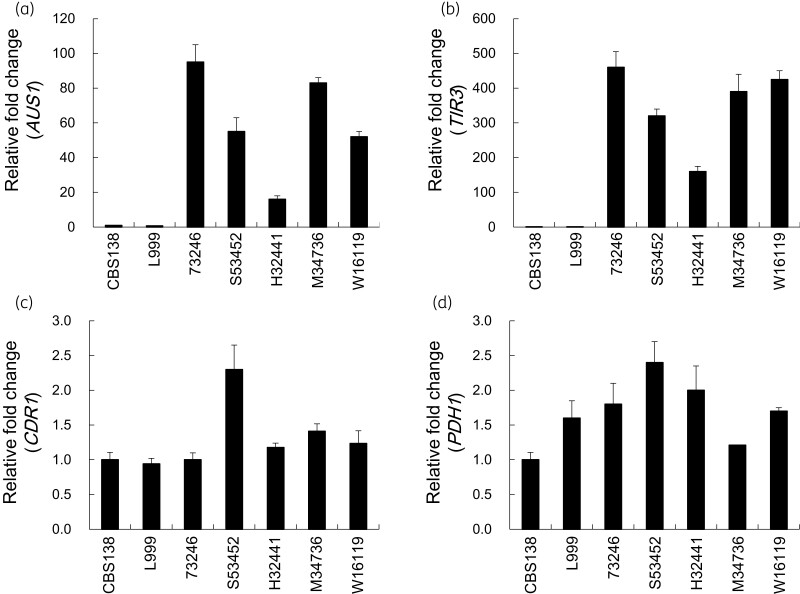
Expression of genes related to either sterol uptake or drug resistance in the sterol-auxotrophic *C. glabrata* strains under aerobic conditions. Total RNA was extracted from aerobically grown *C. glabrata* cells in the presence of 20 mg/L cholesterol. The expression of the genes was evaluated using quantitative RT-PCR analysis with the extracted total RNA. The expression of each gene in the cells is shown as a relative fold change normalized to the expression in CBS138 cells using Livak’s ΔΔCt method. Values are means and standard deviations of triplicate measurements from a representative experiment. (a) *AUS1*, (b) *TIR3*, (c) *CDR1* and (d) *PDH1*.

We had previously demonstrated that both *AUS1* and *TIR3* (which encode an ATP-binding cassette transporter and a mannoprotein, respectively) are indispensable for sterol uptake in *C. glabrata*.^6,9^ The expression level of the genes was kept low in sterol-competent *C. glabrata* cells under aerobic normal growth conditions, whereas the depletion of oxygen or iron resulted in highly enhanced expression of the genes. The expression level of the sterol uptake genes, *AUS1* or *TIR3*, was significantly upregulated in the sterol-auxotrophic strains under aerobic growth conditions (Figure 3). The expression level of *AUS1* or *TIR3* in sterol-auxotrophic mutants was at least 20- or 150-fold of those in CBS138, respectively (Figure 3a and b). These suggest that the two genes responsible for sterol uptake were unusually upregulated under aerobic conditions and made the mutant cells aerobically take up exogenous sterols. The expression of ATP-binding cassette transporter genes, *CDR1* or *PDH1*, which were responsible for enhanced azole resistance, was not significantly upregulated in the sterol-auxotrophic mutants (Figure 3c and d). These results also suggest that enhanced azole resistance in the presence of exogenous sterol was not caused by drug efflux by Cdr1p or Pdh1p (Table 3).

### Sterol-auxotrophic strains harbour mutation(s) in sterol synthesis genes

Sterol profiles of the sterol-auxotrophic *C. glabrata* strains indicated that two of them (W16119 and M34736) were defective in lanosterol synthase (encoded by the *ERG7* gene), whereas the other two (H32441 and S53452) were defective in squalene epoxidase (encoded by the *ERG1* gene).^4^ However, a missense mutation was also detected in *ERG7* of the 73246 genome by next-generation sequencing. To further assess the mutation(s) in the series of sterol synthesis genes, 21 coding region DNA sequence (CDS) of the genes were determined (Table 5). The CDSs of W16119 and M34736 were identical. H32441 and S53452 were also observed to be identical (Table 5). Both W16119 and M34736 shared identical mutations in seven genes (*ERG2*, *ERG7*, *ERG8*, *ERG9*, *ERG10*, *HMG1* and *MVD1*). In contrast, H32441 and S53452 have identical mutations in nine genes (*ERG1*, *ERG2*, *ERG4*, *ERG6*, *ERG7*, *ERG8*, *ERG10*, *ERG24* and *MVD1*). Of all mutations detected, the insertion of five nucleotides (TCGCT) in the *ERG1* gene results in a substitution of amino acid residue and stop codon (I16R followed by a stop codon ‘TAG’) (Table 5). Another single nucleotide deletion in the *ERG7* gene results in Y321T and N322I followed by a stop codon ‘TGA’. The nonsense mutation in *ERG1* or *ERG7* was thought to disrupt the function of the enzyme coded by each gene, whereas the effect from other mutations, which cause amino acid substitutions on the sterol synthesis enzymes, was unclear.

**Table 5.** Mutation(s) detected in the genes responsible for ergosterol synthesis of the sterol-deficient clinical isolates of *C. glabrata*

Gene name	Mutation(s)					
	W16119	M34736	H32441	S53452	73246	L999
*ERG1*			missense^a^	missense^a^		
*ERG2*	I207V	I207V	I207V	I207V	I207V	I207V
*ERG3*						
*ERG4*			T13N	T13N		
*ERG5*						
*ERG6*			R48K	R48K		
*ERG7*	missense^b^	missense^b^	T732A	T732A	missense^c^	
*ERG8*	N448S	N448S	N448S	N448S	N448S	N448S
*ERG9*	C344Y	C344Y				
*ERG10*	N170D	N170D	N170D	N170D	N170D	N170D
*ERG11*						
*ERG12*					F427I	F427I
*ERG13*						
*ERG20*						
*ERG24*			V176L	V176L		
*ERG25*						
*ERG26*						
*ERG27*						
*HMG1*	S92L, K140Q, D775N, G1028A	S92L, K140Q, D775N, G1028A			G1028A, K140Q, D775N	G1028A, K140Q, D775N
*MVD1*	K65N, S70A	K65N, S70A	S70A	S70A	S70A, D362E	S70A, D362E
*IDI1*						

a Missense by insertion (TCGCT) at 45 (15LIV* *→* *15LR*).

b Missense (T961del) (321YNL* *→* *321TI*).

c Missense (C1121del) (374PQGM* *→* *HKV*).

### The sterol-auxotrophic C. glabrata strains retain intact virulence in a mouse model of disseminated infection

**Figure 4.**
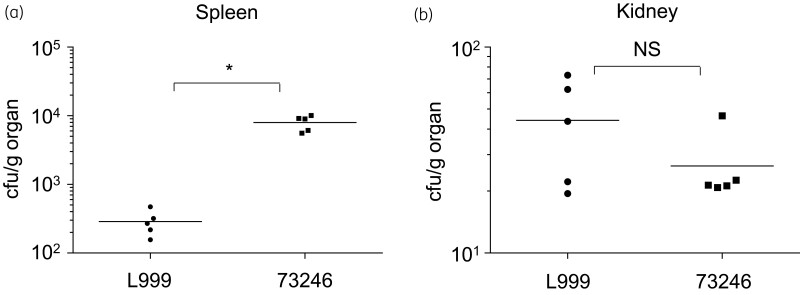
The virulence of sterol-deficient *C. glabrata* with a mouse infection model. Fungal tissue burdens in kidneys (a) and spleen (b) from groups of five BALB/c mice infected via the tail vein with 1* *×* *10^7^ viable cells of the *C. glabrata* strains. Mice were sacrificed at Day 7 post-infection. Results are expressed as cfu/g of tissue and represent values recorded separately for each of the five mice. Geometric means are indicated by horizontal bars. Statistical comparisons are summarized above each panel. Asterisks indicate statistically significant differences (*P <* *0.05; **P <* *0.01). NS indicates no significance (*P *>* *0.05).

The effect of sterol deficiency during disseminated infection was tested in a mouse infection model. Mice were inoculated IV with the sterol-auxotrophic 73246 and its putative parental sterol-competent strain, L999. In mice infected with 73246, the number of cells recovered from spleen was about 28 times higher than that from mice infected with L999 (Figure 4a). However, the cell numbers recovered from the kidney when infected with the two strains were not significantly different from each other (Figure 4b). These results indicate that the defect in ergosterol does not reduce the infection efficacy of *C. glabrata*.

## Discussion

In this study, we evaluated the sterol uptake activity, drug susceptibility and virulence of the sterol-auxotrophic clinical isolates of *C. glabrata*. Our results suggested that the deficiency in sterol synthesis does not attenuate the survival of *C. glabrata* cells inside host bodies. This may be an unacceptable idea for most researchers as sterol synthesis is indispensable for any fungal cells *in vitro*. It was possible that the number of sterol-auxotrophic *C. glabrata* strains inside host tissues was small and that the strains were accidentally isolated under limited clinical situations. These strains were isolated from limited types of specimen (mainly from urine).^5^ However, in continuous surveillance over a 1 month period, 10.8% of the isolates derived from the urine required bile, the source of externally supplied sterol, for growth.^5^ Another trial of isolation of sterol-deficient *C. glabrata* strains revealed that some bile-dependent strains were isolated from the nasopharynx or blood. These accumulated data of clinical isolates indicated that a certain ratio of yeast cells, most of which were *C. glabrata*, exhibited sterol auxotrophy. In yeast, oxygen is required for multiple sterol biosynthesis reactions. Sterol synthesis in *C. glabrata* cells during infections is expected to be restricted as the dissolved molecular oxygen levels are low in some parts of the organs. We demonstrated that the mutant *C. glabrata* strain, which lacked the exogenous sterol uptake (*aus1*Δ), exhibited decreased fungal burden in the mice infection models.^6^ These observations suggest that sterol uptake in *C. glabrata* cells is necessary to obtain enough sterols to grow during infection, and partly illustrate the intact virulence of the sterol-auxotrophic clinical isolates due to the supplementation of exogenous sterols from the host body.

The analysis of cellular sterols in this study revealed that most incorporated sterols in the sterol-auxotrophic strains were retained in the free form, and that the limited ratio of the sterols was esterified (Table S2). The taken-up exogenous sterols were esterified and stored in the lipid particle in *S. cerevisiae*.^17^ The only desirable sterols for growth among the esterified sterols were sorted to the plasma membrane or supplied for the endogenous ergosterol synthesis pathway. The taken-up sterols are also esterified in *C. glabrata* and sorted to plasma membranes as in the case of *S. cerevisiae*.

The amount of total ergosterol in 73246 and S53452 was half or a quarter of the sterol-competent strain, respectively, when ergosterol was exogenously supplied (Figure 2a, Table S2). The growth restoration by ergosterol was comparable to the other sterol-auxotrophic mutants (Figure 1b). These observations suggest that the amount of ergosterol, which probably resides in plasma membranes, did not significantly affect the growth ratio or the physiological membrane property of *C. glabrata* cells, at least within the values for ergosterol detected in this study. The sequencing of ergosterol synthesis genes showed that 73246 and S53452 harbour missense mutations in *ERG7* and *ERG1*, respectively (Table 5). This also indicates that the ergosterol synthesis block point does not correlate with the sterol uptake activities.

The growth restoration by lanosterol, an intermediate compound of the sterol synthesis pathway, was poor in 73246 or M34736 (Figure 1d). The amount of total ergosterol, converted from taken-up lanosterol, in these strains (985* *±* *10 and 482* *±* *20 in 73246 and M34736, respectively) was also slightly smaller than those in other strains (Table S2). This suggests that about 1000 ng/OD_600_ unit of total ergosterol would be required for the intact growth of *C. glabrata* cells. W16119, which harbours a missense mutation in *ERG7* identical to 73246 or M34736, did not show growth restoration by supplementation with lanosterol, whereas similar growth restoration was observed by supplementation with ergosterol or cholesterol (Figure 1b and c). This indicates that W16119 could take up exogenous sterol similar to other strains but had additional mutations in gene(s) other than the missense mutation in *ERG7,* which critically inhibit the conversion of lanosterol to ergosterol. It was also notable that the strain H32441, which harbours a missense mutation in *ERG1*, accumulated a significant amount of squalene. This result did not contradict the previous report that *ERG1*-deleted *S. cerevisiae* mutant accumulate 4-fold the amount of squalene of the WT strain.^18^ S53452 did not show squalene accumulation, however the strain also had a mutated *ERG1* CDS identical to H32441 (Table 5). These results suggest that additional mutation(s) in some genes related to sterol metabolism in S53452 resulted in little squalene accumulation in the strain.

Of 17 commercially available sterol or sterol-related compounds, 10 compounds could support the growth of sterol-auxotrophic clinical isolates of *C. glabrata*. These compounds (group A) were thought to be taken up as efficiently as ergosterol and replaceable with ergosterol at the plasma membrane, as they supported the growth of W16119, which cannot convert lanosterol to ergosterol. It is hard to predict the structural moieties required for the growth supplementation of strains, as there is no clear correlation between the sterols’ structural moiety and growth supplementation. 25-Hydroxycholesterol may not be incorporated into *C. glabrata* cells like other steroidal compounds for unknown reasons or because it is not converted into some functional sterols by *C. glabrata*.

The drug susceptibility of the *C. glabrata* strains reflected the sterol uptake properties of the cells. The MIC of the tested strains for fluconazole was more than the maximum concentration (1024 mg/L) in the presence of any tested sterol-related compounds (Table 3). Fluconazole inhibits the fungal lanosterol 14α-demethylase and inhibits ergosterol synthesis. It was expected that most of the sterol-related compounds taken up exogenously by the *C. glabrata* cells directly became the alternative to ergosterol in the plasma membrane to salvage the growth inhibition by fluconazole. However, it was intriguing to observe that the supplementation with lanosterol also abolished the growth inhibition by fluconazole. The incorporated lanosterol will not be converted into ergosterol in the presence of fluconazole. It was shown that *C. glabrata* cells accumulate an unusual sterol (4,14-demethylzymosterol) when treated with fluconazole, whereas other fungal species accumulate a toxic sterol intermediate in the presence of fluconazole.^11,19^ The characteristic response to fluconazole in *C. glabrata* cells might have enabled the conversion of the supplemented lanosterol to a non-toxic alternative sterol. The MIC of the strains for amphotericin B was intriguing. Amphotericin B is thought to permeabilize the plasma membranes by binding to ergosterol of fungal cells.^20,21^ The MIC of the sterol-auxotrophic mutants for amphotericin B was as much as that of the sterol-competent strains when exogenous ergosterol was supplied (Table 4). Our result suggests that the incorporated ergosterol was sorted to the plasma membrane in the sterol-auxotrophic mutants, and that the major sterol in the plasma membranes was ergosterol. These significant changes in drug susceptibility caused by sterol uptake may partly illustrate the tolerance of *C. glabrata* cells for the drugs targeting ergosterol synthesis during infections.

In the mice infection models, the sterol-auxotrophic strain 73246 exhibited a fungal burden in kidney comparable to that of the putative parental strain L999 (Figure 4b). This suggests that *C. glabrata* cells obtained enough sterol, probably cholesterol from host organs during infections. Interestingly, about 28 times more *C. glabrata* cells were recovered from the spleen in 73246-infected mice when compared with mice infected with sterol-competent L999 (Figure 4a). This further indicates the possibility that auxotrophy for sterol offers a tissue-specific survival advantage to *C. glabrata* cells in some tissues. However, more experiments with other sterol-auxotrophic strains should be conducted to make such a proposition. It is also suggested that a mouse mortality model or *Galleria mellonella* larval model will give a clear answer to the role of sterol uptake in virulence.

The results in this study and previous reports demonstrated the significance of sterol uptake of *C. glabrata* cells in both virulence and drug susceptibility inside host tissues. More attention should be paid to this characteristic activity of *C. glabrata* under clinical situations.

## Supplementary Material

dlac018_Supplementary_Data

## Supplementary data

Tables S1 and S2 are available as Supplementary data at *JAC-AMR* Online.
