# Supplementary material for: Exhibition of antifungal resistance by sterol-auxotrophic strains of Candida glabrata with intact virulence
Source: JAC Antimicrob Resist. 2022 Mar 7;4(1):dlac018. doi: 10.1093/jacamr/dlac018 (PMC8900151; doi:10.1093/jacamr/dlac018)
Supplement: dlac018_Supplementary_Data [file dlac018_supplementary_data.docx]

**Supplementary data**

**Table S1**. Primers used in this study

| <For amplification of *ERG* genes> |  |
| --- | --- |
| Primer name | Sequence (5' → 3') |
| ERG1-653f | GTCCGTAAACGACTAGCGACTCTTT |
| ERG1 802r | ATCCTTCTGTGCCTCTTCTGCACGA |
| ERG2-643f | GCCATATCATCCTGTTTGCTCGGAG |
| ERG2 530r | AGAATGACGATGATGAAGCGGAGGA |
| ERG3-334f | TATACGAGTGTGCTCTTTGCGGATC |
| ERG3 663r | TTATATGCCTCTGTGGAGGCGAGGA |
| ERG4-510f | TCGGCGTATGCGTTGAATGTAATCC |
| ERG4 761r | AGGGTGAAGCAGCTGAGTTTCGTTA |
| ERG5-395f | GTCACATGACGTATTCACACACGGA |
| ERG5 872r | TTATGGTTGGCTACGTCAGTGGAGG |
| ERG6-82f | TGTGCTCTTGACGTCTCTAAAGCGT |
| ERG6 103r | ACGACAGGTAACACTCATAAGGCCA |
| ERG7-653f | GGAGCATTGCTATAATGGAACGCTG |
| ERG7 568r | ATATACAGATTGTGGAGTAGGCCCC |
| ERG8-379f | TAAACCCTTCTATTGGGGAGGGGAC |
| ERG8 880r | ATGACGGGTCTACCCTCATAACAGA |
| ERG9-460f | AGTACTACAAGCTGGCCATTGGCAT |
| ERG9 437r | TAGCGAGCTCAGCAAGAAGTTTGAC |
| ERG10-234f | AACCGTACCAACACATCGGAGATAG |
| ERG10 450r | CTGGCGGCTTCTGGTACTAATTTAC |
| ERG11-398f | ATCCAGTTGCACACATCGTATACCC |
| ERG11 865r | GGCAATACCTCAATGAGGCCACAAA |
| ERG12-266f | GTGTAAAGTAGTGGTTGCAGCACCT |
| ERG12 75r | AACCGATTACGAGGGCCTGTACCAA |
| ERG13-591f | TTCTCGATGAGTGAATGGGAGGAAG |
| ERG13 392r | CGGTTCGTGCCGAAACTATGTCACT |
| ERG20-542f | TCTTCAAGACGTGGGTGGGCTTAGT |
| ERG20 655r | CGGAATCTATGGTGTTCTTCCCTGG |
| ERG24-498f | CTATCTTGCCTTGCACCTCAATGCT |
| ERG24 598r | TCGGCTCAGTGGAGATTCCAGAGTT |
| ERG25-306f | GAAAGGCTAGGATGGGCTCGAAGTA |
| ERG25 535r | CTATGATGCCTCACCCTGAGAGAGT |
| ERG26-292f | CAGTTGTTGCAGCTATAGCGTACCA |
| ERG26 671r | ATTAAATGGGGCGAGCTGGGAATTG |
| ERG27-556f | CCAGTGATGAAGCTGGCTAAGGACA |
| ERG27 866r | ACAATGCCACGCCATTGAAAAGACC |
| HMG1-692f | CTATCAGGCATCTCAGCATCTATCG |
| HMG1 913r | TTGAAAGAGTGTCAGTCTCCCAGGA |
| MVD1-659f | CAGAGATGCAGAGGTTTCCATAGCG |
| MVD1 961r | TCTCCGGTTCCTTGGACAGATCTGT |
| IDI1-516f | TCTGCTATGTAGGTTTAGAGCCGCG |
| IDI1 432r | GGTACGATGCTAAATAAGCCCGAAC |
|  |  |
| For sequencing |  |
| Primer name | Sequence (5' → 3') |
| ERG1-1 | ATGTCTCTCACGAATGCT |
| ERG1-301 | CTCGACTGGCTTCAAGTC |
| ERG1-601 | TGTGACGGTATCTTCTCC |
| ERG1-1201 | AACTTGAAGGCTCTACAA |
| ERG2-5 | AGTTCTTTATCAATCTGC |
| ERG2-301 | TTCAGAAATGGAAGCATG |
| ERG3-1 | ATGGATTTGGTGTTGGAA |
| ERG3-304 | TAGCAGACCGAAGACGGT |
| ERG3-614 | ACTGGCCTCGTGTCTACA |
| ERG4-1 | ATGAGCGGATCGAAAGAA |
| ERG4-301 | GAAGAAGATTTGGAAAGC |
| ERG4-602 | ATGATTTCTTTATGGGAG |
| ERG4-1201 | TGGACCCAATCTTTGATC |
| ERG5-1 | ATGGCTTCTGTAGTAGAT |
| ERG5-302 | TGGAGACACAGGACAATG |
| ERG5-601 | CCACAGATCTTCTTCCAT |
| ERG5-1201 | TTGATGGTCCCATATGTC |
| ERG6-1 | ATGTCTGAGTCGGAATTG |
| ERG6-309 | TGTTCGTGACGAGCCATG |
| ERG6-601 | CCAAAGCTGGAAGGTGTA |
| ERG7-2 | TGACTCAGTTTTATTCTG |
| ERG7-301 | CCCAATTGTCATGAACAT |
| ERG7-602 | ACTTATATAAATGGGAGG |
| ERG7-1203 | CTTGGCTGAGAAACCGGA |
| ERG7-1801 | CACTCTGTAGGAGAAACC |
| ERG8-2 | TGAAAAACATGGAAAGAG |
| ERG8-301 | ACTAGCAGGTCCTATAAA |
| ERG8-601 | ATCGGCAGTGGGTTTGAT |
| ERG8-1201 | GCAGTATCCATTATCTCC |
| ERG9-1 | ATGGGTAAAGTACTTGAC |
| ERG9-301 | GGTGGTGTCCAACTTGGA |
| ERG9-601 | TCTGACGATTTATACCAT |
| ERG9-1201 | TTGAAGGTCCAAGAAAGA |
| ERG10-1 | ATGAGTGATACTGTCTAC |
| ERG10-302 | ACTTGATGGCTTGGGCAC |
| ERG10-601 | GAAATCGTTCCAGTTACC |
| ERG11-1 | ATGTCCACTGAAAACACT |
| ERG11-301 | ACCCAAGTAGACAGTCAT |
| ERG11-601 | CAACCTGAAATGACTATC |
| ERG11-1204 | CCAAGGGACTACCACGTT |
| ERG12-1 | ATGACTGTAGAGCGGGAC |
| ERG12-301 | CAGAATAACATTCAGCAG |
| ERG12-601 | GGTATAGACAATGCGGTA |
| ERG12-1201 | CCGCTGTTCGAAAATACT |
| ERG13-1 | ATGACTGAGATTAAGAAG |
| ERG13-301 | AACAGACTTGGACTTATC |
| ERG13-601 | TACGATTTCTACAAGCCA |
| ERG13-1201 | AACAAGCTACAAAGCAGA |
| ERG20-1 | ATGTCAAAAGAAGCAAGT |
| ERG20-301 | AATGGACTTGTCCATCAT |
| ERG20-601 | TCCTTTTATCTTCCAGTT |
| ERG25-1 | ATGTCTGCCGTTTTCAAC |
| ERG25-301 | CAGGACGGCCTTCAAACA |
| ERG25-602 | GTACCGTCGGTATGCCAA |
| ERG26-1 | ATGTCTGAAATCAAGTCT |
| ERG26-307 | CAATAATATTTCTGGTACC |
| ERG26-601 | GGTCAGTCAAAGTTTCAA |
| ERG27-1 | ATGACATCCAAGACCAGG |
| ERG27-301 | CTGTGCAGCATTCACAAA |
| ERG27-601 | GTTACTTATGAAGGTTCT |
| HMG1-1 | ATGCCTTTACTTTTCGAG |
| HMG1-302 | GCAGATAATAATGTTGAG |
| HMG1-601 | ATGATGTACACCATCTTC |
| HMG1-1210 | TATGCTGTTGTATTTGTC |
| HMG1-1801 | CTGGAAGCTATAATGAAG |
| HMG1-2401 | ACTACAACTGGTGATGCG |
| HMG1-3001 | GAACTGTCGCTGTGTGCG |
| MVD1-1 | ATGACATACGTTGCTTCT |
| MVD1-301 | GACAATGTGAAGCTTCCA |
| MVD1-601 | GACAAGAAGGATACACCA |
| IDI1-1 | ATGTCAGAATACAGGAAG |
| IDI1-301 | CAATAGTTTACCCTCAGT |
| IDI1-601 | TACAAGATCGATGAAGGT |
| ERG24-1 | ATGTTGAATCCCAAGACT |
| ERG24-301 | GTACTTCAACTTAGTGCC |
| ERG24-601 | GAGTTGAACCCTCGCATC |
| ERG24-1201 | TTGCTGCTGCATAGACAA |
| <For semiquantitative PCR> |  |
| Primer name | Sequence (5' → 3') |
| CgAUS1-F | AGTGTCCCCTTGCGAAAATG |
| CgAUS1-R | GCACCGAGCACTAGCACCAT |
| CgTIR3-F | CGCTGAAAACGATCCAAACTTC |
| CgTIR3-R | TGGTCATAGCCATGTATAGATCCAA |
| CgCDR1-F | GCTTGCCCGCACATTGA |
| CgCDR1-R | CCTCAGGCAGAGTGTGTTCTTTC |
| CgPDH1-F | GCCATGGTACCTGCATCGAT |
| CgPDH1-R | CCGAGGAATAGCAAAACCAGTATAC |
| Cg18S-F | AATGCACCCGGGCCTTT |
| Cg18S-R | GCCGCCAAGCCACAAG |

**Table S2**. The cellular sterol contents of the sterol-deficient clinical isolates of *Candida glabrata*

| strain | sup | Sterol content (ng/OD600 unit) | | | | | | |
| --- | --- | --- | --- | --- | --- | --- | --- | --- |
|  |  | Erg(t) | Erg(f) | Chol(t) | Chol(f) | Lano(t) | Lano(f) | squalene |
| CBS  138 | NC | 3211±52 | 2901±120 | N.D. | N.D. | 128±12 | 110±15 | 87±8 |
|  | Erg | 2933±101 | 2752±41 | N.D. | N.D. | 83±9 | 80±7 | 6±0 |
|  | Chol | 2852±73 | 2533±63 | N.D. | N.D. | 111±11 | 91±7 | 47±3 |
|  | Lano | 1902±16 | 1855±65 | N.D. | N.D. | 68±2 | 135±15 | 42±3 |
| L999 | NC | 2452±82 | 2401±155 | N.D. | N.D. | 35±1 | 31±2 | N.D. |
|  | Erg | 2483±63 | 2414±24 | N.D. | N.D. | 32±1 | 31±1 | N.D. |
|  | Chol | 3086±18 | 2888±96 | N.D. | N.D. | 41±2 | 33±2 | N.D. |
|  | Lano | 2321±41 | 2147±122 | N.D. | N.D. | 29±1 | 39±5 | N.D. |
| 73246 | Erg | 1025±43 | 851±32 | N.D. | N.D. | N.D. | 12±1 | N.D. |
|  | Chol | N.D. | N.D. | 2335±42 | 2185±152 | N.D. | N.D. | N.D. |
|  | Lano | 985±10 | 692±9 | N.D. | N.D. | 110±6 | 83±5 | N.D. |
| S | Erg | 582±14 | 555±15 | N.D. | N.D. | 8±1 | 5±1 | 110±8 |
|  | Chol | N.D. | N.D. | 1390±36 | 1402±80 | N.D. | N.D. | 52±7 |
|  | Lano | 1601±75 | 1321±74 | N.D. | N.D. | 41±2 | 39±2 | 78±6 |
| H | Erg | 2546±103 | 2156±152 | N.D. | N.D. | N.D. | N.D. | 3802±122 |
|  | Chol | N.D. | N.D. | 1824±85 | 1745±45 | N.D. | N.D. | 1084±41 |
|  | Lano | 1204±124 | 1280±94 | N.D. | N.D. | N.D. | 4±0 | 1022±25 |
| M | Erg | 2141±74 | 1855±102 | N.D. | N.D. | N.D. | N.D. | N.D. |
|  | Chol | N.D. | N.D. | 1301±70 | 1188±21 | N.D. | N.D. | N.D. |
|  | Lano | 482±20 | 442±6 | N.D. | N.D. | 62±3 | 53±3 | N.D. |
| W | Erg | 2412±223 | 2368±7 | N.D. | N.D. | 15±1 | 7±1 | N.D. |
|  | Chol | N.D. | N.D. | 1845±56 | 1622±74 | N.D. | N.D. | N.D. |

Values represent means and standard deviation of triplicate measurements from a representative experiment. N.D.; Not detected.

Strain names: S; S53452, H; H32441, M; M34736, W; W16119

Sup (supplement): NC; ethanol+Tween80, Erg; ergosterol, Chol; cholesterol, Lano; lanosterol

Sterol content: Erg(t); ergosterol (total), Erg(f); ergosterol (free), Chol(t); cholesterol (total), Chol(f); cholesterol (free), Lano(t); lanosterol (total), Lano(f); lanosterol (free)
